# Following hearts, one cell at a time: recent applications of single-cell RNA sequencing to the understanding of heart disease

**DOI:** 10.1038/s41467-018-06894-8

**Published:** 2018-10-30

**Authors:** Matthew Ackers-Johnson, Wilson Lek Wen Tan, Roger Sik-Yin Foo

**Affiliations:** 1 Cardiovascular Research Institute, National University Health System, 14 Medical Drive, Singapore, 117599 Singapore; 2 Genome Institute of Singapore, 60 Biopolis Street, Singapore, 1368672 Singapore

## Abstract

The mammalian heart contains heterogeneous cell types contributing to pathological changes in cardiac disease. In this Comment, we explore how single-cell transcriptomic approaches are unveiling intricate cellular mechanisms and gene co-expression networks that regulate the workings, and failings, of the heart.

## Introduction

The mammalian heart is a complex organ, comprising a precise but heterogeneous arrangement of specialised myocytes and non-myocytes, the function of and dynamic interplay between which are critical in determining cardiac health and pathophysiological processes. In past decades, traditional approaches to study cardiac biology using whole tissue or bulk cell isolates have offered great insight, but are nonetheless limited in resolution, thus biologically relevant information may be obscured.

Single-cell RNA sequencing (scRNA-seq) offers the ability to investigate global transcriptomic profiles of individual cells, the most basic units of life. Since its inception less than 10 years ago^1^, scRNA-seq technologies and applications, accompanied by customised bioinformatic analysis tools, have diversified and expanded rapidly, with ever increasing sensitivity, accuracy and throughput, coupled with decreasing costs and labour demands^2,3^. Typical data processing of scRNA-seq involves quality control, normalisation, confounding factor identification and in-depth analysis. According to the research question, investigators may currently select between techniques permitting detailed transcriptomic analysis of hundreds of cells in high transcriptomic detail, or pursue the analysis of tens of thousands of cells at reduced sequencing depth^4,5^.

The advent of scRNA-seq has afforded cardiac researchers the opportunity to examine heart biology at unprecedented cellular resolution, and importantly, without reliance on cell surface or genetic lineage tracing markers. Such markers are typically imperfect and may conceal heterogeneity that exists, even within populations of what are believed to be similar cell types. However, an immediate technical hurdle encountered is the unique difficulty of dissociating the adult mammalian heart tissue without damaging constituent cells, particularly cardiomyocytes^6^. This, in addition to their large and irregular shape, precludes investigation by most standard single-cell techniques. Perhaps partly for this reason, alongside keen interest in regenerative potential and congenital heart disorders, initial scRNA-seq studies focused on embryonic and neonatal murine hearts and adult zebrafish hearts^7–9^. These studies yielded exquisite temporal and anatomically defined transcriptomic maps of major cell types during normal and abnormal cardiac development, and a novel regulator of cardiac regeneration, respectively.

## Recent advances in the study of the adult mammalian heart

Recent studies have dramatically expanded the scope of scRNA-seq research into adult mammalian hearts. To circumvent the problem of cardiomyocyte dissociation, See et al.^10^ adapted an established microfluidic platform for single nuclear RNA-seq (snRNA-seq). Nuclei are less fragile than their cellular counterparts, and can be isolated even from frozen, banked tissue. snRNA-seq of mouse and human failing and non-failing adult hearts revealed considerable cellular heterogeneity, and the presence of cardiomyocyte sub-populations with altered expression of cell-cycle related genes. Although cytoplasmic RNA is lost, snRNA-seq allows the capture of RNA undergoing nascent transcription, in a snap-shot of the in vivo setting at point of harvest. Nuclear transcripts may also be enriched for non-coding RNA species, and indeed, two novel lincRNAs with potential cell-cycle regulatory roles were identified, which do not present themselves when analysing contemporaneous bulk, or whole-cellular, RNA-seq data^10^.

Cardiac non-myocyte cells outnumber myocytes in the adult mammalian heart, and critical roles of these cells in both healthy cardiac homoeostasis and disease are becoming increasingly evident. Two recent studies have foregone efforts to retain viable cardiomyocytes, focusing instead on global identification and characterisation of adult mouse heart non-myocyte populations, using a commercial, high throughput nanodroplet-based scRNA-seq platform and kit (Chromium; 10× Genomics)^11,12^. Comparative analysis of thousands of cells in each case, although sequenced at relatively low-read depth, enabled clustering and transcriptome-informed identification of multiple cardiac-resident non-myocyte cell-types and sub-types. Schafer et al.^11^ employed a fibrotic disease model and utilise data specifically to elucidate the cellular sub-population responsible for production of a newly discovered fibrotic cytokine. Skelly et al.^12^, however, proceed to perform comprehensive analysis of diverse non-myocyte populations in healthy mouse heart, revealing novel marker gene expression signatures, rare cell types and putative intercellular communication networks based on cell-type specific expression of ligands and cognate receptors. A combination of parameters including total number of unique genes expressed, unique molecular identifier counts and fraction of mitochondrial-mapped reads, were employed for quality control.

It is interesting to note that in the previous two studies, using simple enzymatic dissociation of ventricular tissue, almost no viable cardiomyocytes are identified, in line with our expectations^6^. A subsequent cardiac scRNA-seq study, by Gladka et al.^13^, is therefore surprising, because using similar methodology, the authors report high yields of viable myocytes, as well as non-myocytes. A tailored, fluorescence-activated cell sorting (FACS)-based approach is applied to sort a combined 1000 cells from healthy myocardium and diseased myocardium following surgically induced ischaemic injury. Subsequent scRNA-seq and analysis of transcriptomic data permit clustering and identification of cellular populations and sub-populations, of which some are disease-enriched, and all of which exhibit cellular heterogeneity, information that could not have been resolved by bulk sequencing. Further investigation of fibroblast disease-associated sub-populations led to the identification and validation of a novel marker and putative regulator of activated cardiac fibroblasts, *Ckap4*^13^.

How might one explain the myocyte discrepancy between these studies? Gladka et al.^13^ assume that all cellular particles negative for DAPI nuclear staining are viable. However, the myocytes shown in figures appear fragmented, and DAPI-negative particles could conceivably include myocyte fragments that do not contain or have lost their nucleus during dissociation. Ideally, the viability assay would employ both a positive, cell-permeable stain to confirm the presence of a nucleus, in addition to a non-permeable dye that viable cells should exclude^12^. We also note the high mitochondrial read fractions in myocytes (80%), fibroblasts and endothelial cells (50%), which may further allude to the capture and sequencing of compromised cells. Nonetheless, a tailored, flexible FACS-based approach to derive biologically meaningful cardiac scRNA-seq data from relatively few cells^13^, is a welcome contribution to the field.

A new study in Nature Communications applies scRNA-seq to convincingly viable and intact adult mammalian myocytes^14^. Nomura et al. successfully combine established Langendorff-perfusion and chunk-based mouse and human ventricular dissociation methods, respectively, with downstream single-cell cDNA library construction, by manual micro-pipetting of live cardiomyocytes into lysis buffer. Integrated transcriptomic, morphological and epigenetic analysis subsequently identifies cell state trajectories, and functionally implicates a critical p53/Mef2/Nrf2 signalling axis at the transition to heart failure in a mouse pressure overload model, which is furthermore conserved in failing human hearts. This demonstration of scRNA-seq using isolated adult myocytes is an exciting development and holds promise as a basis for future studies.

## Future directions

Forthcoming research is likely to trend towards studies achieving greater mRNA capture rates and sensitivity, with numbers of cells that are orders of magnitude higher than currently employed. Rationale for this approach in cardiac research includes the identification of increasingly rare cell populations, such as progenitor cells and immunocyte sub-types, with sufficient sensitivity and depth to permit thorough molecular characterisation and elucidation of gene regulatory networks. Cellular biology and intercellular communication relationships may be further explored in multiple mammalian disease models and human cardiac pathologies and across multiple timepoints, leading to advanced understanding of mechanisms in heart disease and novel therapeutic targets.

Emerging multiplexed sequencing methods such as single-cell combinatorial indexing RNA sequencing and split pool ligation-based transcriptome sequencing have potential to increase throughput to millions of cells. Single-cell universal poly(A)-independent RNA sequencing and multiple annealing and dC-tailing–based quantitative single-cell RNA sequencing may afford exciting opportunities to include non-poly-adenylated RNA species such as circular RNAs, which have emerging roles in cardiac biology^3,15^. Meanwhile, Nanopore sequencing platforms may further revolutionise scRNA-seq capabilities, and combinatorial approaches could allow single-cell analyses of protein, RNA and DNA. For example, “scTrio”-seq aims to define relationships between genomic copy-number variants, the DNA methylome, and the transcriptome, of the same mammalian cell^2^.

The availability and dissociation of viable myocyte and non-myocyte cells from fresh tissue, particularly human, continues to hinder scRNA-seq studies in the cardiac arena. Adult cardiomyocytes are too large to fit into most commercial single-cell microfluidic chambers, and do not generally tolerate FACS well. snRNA-seq is one option, but lacks cytoplasmic RNAs. Manual micro-pipetting is laborious and difficult to upscale. Alternative solutions may be found in techniques such as laser capture microscopy (LCM)-seq^16^, and fluorescent in situ (FIS)-seq^17^. LCM-seq involves direct selection and excision-capture of cells in histological slices, which are then subjected to routine scRNA-seq pipelines. In contrast, FIS-seq profiles gene expression directly on tissue sections in situ, using confocal image-based analysis. While labour-intensive, both techniques do not require cell dissociation, may be applied to frozen, stored specimens, and importantly, preserve spatial anatomical information.

## Conclusions

The extraordinary resolution and rich data of scRNA sequencing studies discussed here, and others (see Table 1), continue to drive critical insight into cardiac biology, in health and in disease. Improved understanding of congenital defects in heart development^7,8^, regenerative capacity^9,10^, cytokine signalling^11^, rare cell types plus autocrine and paracrine intercellular communication networks^12^ and new endogenous markers and mechanistic regulators of disease-associated processes^13,14^, all point naturally to novel targets for future diagnostic and/or therapeutic intervention strategies. As technologies continue to improve, costs decrease and user-friendly commercial kits, platforms and bioinformatic analysis suites become mainstream, accessibility and progress in both research and clinical personalised medicine applications for scRNA-seq are an ever-closer reality^2,3,5^.

**Table 1:** Single-cell cardiac RNA-seq analyses

Publications		Species	Tissue	Context	Total cells used in analysis
Kokkinopoulus et al. (2015)	PLoS ONE	Mouse	Embryonic hearts	Early allantoic bud, late allantoic bud, and early headfold stages	1088 cells
Cao et al. (2016)	Development	Zebrafish	Adult epicardial cells	Epicardial cells from *tcf21*-reporter labelled uninjured adult hearts	39 cells
DeLaughter et al. (2016)	Dev. Cell	Mouse	Developing hearts	7 time-points spanning E9.5 to P21; left atrium, primodial ventricle, left and right ventricles	>1200 cells
Li et al. (2016)	Dev. Cell	Mouse	Embryonic hearts	Anatomical zones of E8.5, E9.5 and E10.5 hearts	2233 cells
See et al. (2017)	Nat. Commun.	Mouse, human	Adult left ventricles	Sham & TAC (mice); Non-failing & DCM (human)	359 nuclei (mouse); 116 (human)
Liu et al. (2017)	Nature	Mouse	iCM	D3 cardiac fibroblasts infected with *Mef2c*, *Gata4* and *Tbx5* viral constructs	513 cells
Schafer et al. (2017)	Nature	Mouse	Adult hearts	Hearts from *Pln*^R9C/+^ mice	1263 cells
Skelly et al. (2018)	Cell. Rep.	Mouse	Adult left ventricles	Non-myocyte cells from ventricles of male and female mice	10,519 cells
Gladka et al. (2018)	Circulation	Mouse	Adult left ventricles	Control and ischaemic-injured hearts (3d post-injury)	932 cells
Lescroart et al. (2018)	Science	Mouse	Embryonic hearts	E6.75 and E7.25 *Mesp1*-labelled cardiac progenitors	598 cells
Sereti et al. (2018)	Nat. Commun.	Mouse	Embryonic hearts	E9.5, E12.5 and P1 from *αMHC*-GFP murine hearts	122 cells
Xiao et al. (2018)	Dev. Cell	Mouse	Embryonic hearts	E13.5 and E14,5 hearts from control and *Lats1/2* CKO mice	18,166 cells
Su et al. (2018)	Nature	Mouse	Coronary vascular wall cells	Vascular lineage labelled cells	1152 cells
Spanjaard et al. (2018)	Nat. Biotechnol.	Mouse	Larvae and adult heart, liver, pancreas, telencephalon	Developmental lineage tracing using genetic scars	112,322 cells
Nomura et al. (2018)	Nat. Commun.	Mouse, human	Adult hearts	8-week transition to heart failure in a TAC model (mouse); non-failing and DCM (human)	396 cells (mouse); 411 (human)

*TAC*, transverse aortic constriction; *DCM*, dilated cardiomyopathy; *iCM*, induced cardiomyocytes; *CKO*, conditional knockout.
